# Risk of tuberculosis in patients with diabetes: population based cohort study using the UK Clinical Practice Research Datalink

**DOI:** 10.1186/s12916-015-0381-9

**Published:** 2015-06-05

**Authors:** Louise Pealing, Kevin Wing, Rohini Mathur, David Prieto-Merino, Liam Smeeth, David A. J. Moore

**Affiliations:** Faculty of Infectious and Tropical Diseases, London School of Hygiene and Tropical Medicine, London, WC1E 7HT UK; TB Centre, London School of Hygiene and Tropical Medicine, London, WC1E 7HT UK; Faculty of Epidemiology and Population Health, London School of Hygiene and Tropical Medicine, London, WC1E 7HT UK

**Keywords:** Tuberculosis, Diabetes, CPRD, Epidemiology, Cohort

## Abstract

**Background:**

Previous cohort studies demonstrate diabetes as a risk factor for tuberculosis (TB) disease. Public Health England has identified improved TB control as a priority area and has proposed a primary care-based screening program for latent TB.

We investigated the association between diabetes and risk of tuberculosis in a UK General Practice cohort in order to identify potential high-risk groups appropriate for latent TB screening.

**Methods:**

Using data from the UK Clinical Practice Research Datalink we constructed a cohort of patients with incident diabetes. We included 222,731 patients with diabetes diagnosed from 1990–2013 and 1,218,616 controls without diabetes at index date who were matched for age, sex and general practice. The effect of diabetes was explored using a Poisson analysis adjusted for age, ethnicity, body mass index, socioeconomic status, alcohol intake and smoking. We explored the effects of age, diabetes duration and severity. The effects of diabetes on risk of incident TB were explored across strata of chronic disease care defined by cholesterol and blood pressure measurement and influenza vaccination rates.

**Results:**

During just under 7 million person-years of follow-up, 969 cases of TB were identified. The incidence of TB was higher amongst patients with diabetes compared with the unexposed group: 16.2 and 13.5 cases per 100,000 person-years, respectively. After adjustment for potential confounders the association between diabetes and TB remained (adjusted RR 1.30, 95 % CI 1.01 to 1.67, *P* = 0.04). There was no evidence that age, time since diagnosis and severity of diabetes affected the association between diabetes and TB. Diabetes patients with the lowest and highest rates of chronic disease management had a higher risk of TB (*P* <0.001 for all comparisons).

**Conclusions:**

Diabetes as an independent risk factor is associated with only a modest overall increased risk of TB in our UK General Practice cohort and is unlikely to be sufficient cause to screen for latent TB. Across different consulting patterns, diabetes patients accessing the least amount of chronic disease care are at highest risk for TB.

**Electronic supplementary material:**

The online version of this article (doi:10.1186/s12916-015-0381-9) contains supplementary material, which is available to authorized users.

## Background

In the UK, rising rates of tuberculosis (TB), particularly in socially disadvantaged groups including migrants, are of great public health concern. London has the highest TB incidence of any western European capital [1]. There is evidence that reactivation of latent disease accounts for a large number of new TB diagnoses in the UK providing an argument for screening and treating latent disease in high-risk groups to help reverse the UK’s increasing incidence of TB [1, 2]. An effective TB control program that targets latent disease within a UK population will need to define high-risk groups and strategies for accessing and screening them. It could be that the role of diabetes as an independent risk factor for TB disease, or identification of subgroups of diabetic patients at particularly high risk, might inform any future UK screening and treatment policy.

Previous cohort studies have demonstrated an increased risk for TB disease in people with diabetes. The most recent meta-analysis that included three cohort studies showed a rate ratio of 3.11 (95 % confidence interval (CI) 2.27 to 4.26) for pulmonary TB associated with diabetes [3]. None of these studies were based in a UK population and two of the studies were in patients with renal failure, which is an independent risk factor. There has been one previous cohort study in a UK population [4] using two linked hospital based datasets from Oxford which found rate ratios of 1.83 (CI 1.26 to 2.60) and 3.11 (CI 1.17 to 7.03) for active TB disease comparing patients with diabetes with a reference hospital cohort. There has been no previous large cohort study in the general UK population exploring the association between diabetes and TB risk, which has also been able to include the effects of potentially important confounders.

We used a large UK representative general practice database to assess the overall risk of TB comparing people with and without diabetes and to explore whether factors relating to the patient, diabetes condition or accessing primary health care further defined a high risk group for TB, whilst adjusting for important confounding risk factors.

## Methods

We undertook a matched cohort study to investigate the incidence of TB in patients with and without diabetes.

### Clinical Practice Research Datalink

The UK Clinical Practice Research Datalink (CPRD) contains anonymised data from clinical general practice records of more than 15 million patients. Data on demographic, diagnostic, prescription, referral, clinical test results and pertinent lifestyle measures are included in the records. Data collection started in 1987 and currently the database contains information from the medical records of 680 UK participating general practices where there are 7 million patients currently registered. The information contained on the database undergoes regular rigorous quality checks.

### Hospital episode statistics

Hospital Episode Statistics (HES) data include information on English NHS Trust hospital admissions from 1989 and outpatient clinic attendances from 2003. Key diagnostic (using international classification of diseases codes, ICD-10) and demographic data are contained in this database and hospital data for patients residing within the English region of the CPRD are eligible to be linked with their CPRD general practice record. We used HES ethnicity data.

### Study participants

**Fig. 1:**
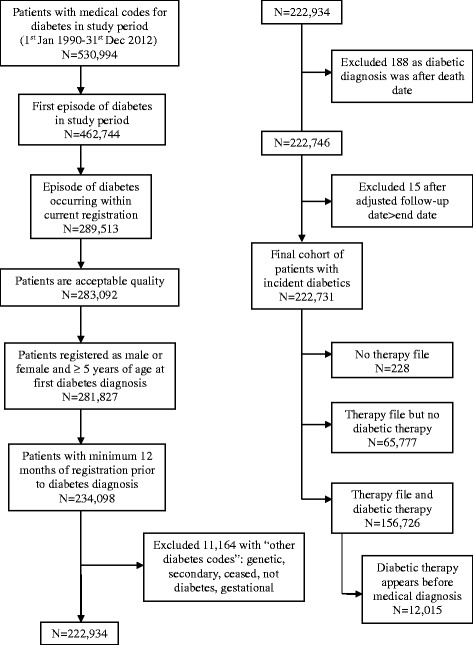
Flow chart of patients with incident diabetes included in the study

We identified a cohort of patients within the CPRD with incident diabetes (types 1 and 2 included), ≥5-years old, who had their first recorded diagnosis for diabetes in the study period 1 January 1990 to 31 December 2012. Patients were identified using National Health Service (NHS) Read codes, which are a coded thesaurus of clinical terms and diagnoses [5]. A diagnosis of diabetes was considered incident if the Read code was first recorded on a date at least 12 months after the patient’s current practice registration date, to avoid including prevalent cases recorded when patients register [6]. The incident date was taken as the date of the first diabetes diagnostic Read code. Patients were excluded if they had a secondary, gestational or genetic cause of diabetes recorded or had codes indicating the earlier diabetes Read code was incorrect (example “ceased” or “not” codes). Once this initial cohort of patients with incident diabetes was created, further data quality checks were made excluding those who did not have a feasible temporal sequence for dates of birth, registration and death (Fig. 1).

The cohort of patients identified with incident diabetes was initially divided into type 1 and 2 diabetes containing *definite, probable* and *possible* categories based on their diagnostic Read code and number of contradictory codes according to methods previously published by De Lusignan et al. [7] The cohorts were then further refined using algorithms including patient age at diagnosis, prescriptions for insulin and other anti-diabetic drugs (OAD), body mass index (BMI) and ethnicity data, where this was available, based on methods first described in the Royal College of General Practitioners’ report of coding and classifying diabetes [8] (Additional files 1 and 2).

**Fig. 2:**
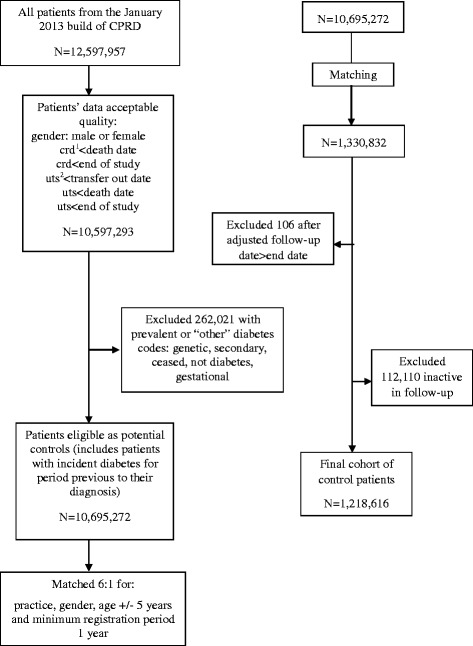
Flow chart of unexposed control patients included in the study. Crd^1^: current registration date in practice. Uts^2^: practice data up to standard quality

An unexposed matched cohort was created, composed of those who did not have a prevalent diagnosis of diabetes on the matched index date, which also had to fall 12 months on or after their current registration date. Up to six unexposed patients were randomly selected and matched for age +/− 5 years, gender and General Practice with every exposed patient. Patients in the unexposed group could become diabetic during the study period and would then join the exposed cohort for the remainder of their follow-up. This was done so that our exposed cohort was not compared against an unusually healthy unexposed group who were never at risk of diabetes. This design helps control for unmeasured confounding lifestyle factors and allows for time dependent analyses. Patients included in the unexposed cohort had to have at least one face-to-face consultation or acute prescription recorded within the 12 months before or after their matched index date to ensure they were active in their follow-up. The unexposed cohort was subject to the same data quality checks and exclusions as the exposed cohort (Fig. 2).

### Tuberculosis outcome

**Fig. 3:**
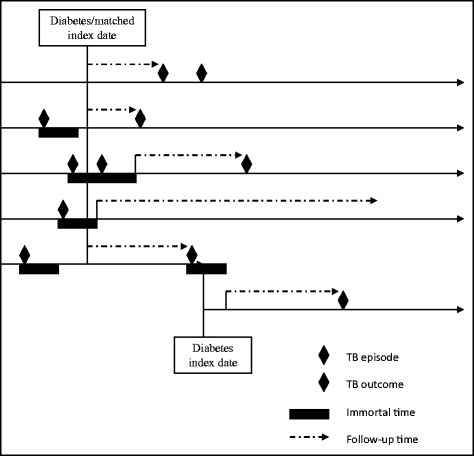
Assignment of follow-up time allowing for previous tuberculosis diagnosis

A list of Read codes for all forms of tuberculosis was developed (available on request). Prescriptions for anti-tuberculosis drugs was not used in developing or later validating cases of TB identified by diagnostic Read codes as these medications singly prescribed are not specific to active TB disease which is treated by secondary care specialists in the UK. Both exposed and unexposed patients were allowed to have had TB recorded before their index or matched index date but there was a lag period of 2 years after the last coding of TB before a subsequent coding of TB would be considered as a new diagnosis. Any part of this lag period which occurred after the (matched) index date was not included as follow-up time in order to avoid time-related bias [9] (see Fig. 3).

### Confounders and effect modifiers

We considered potential confounders including age, gender, BMI, alcohol intake, smoking status, socioeconomic status and ethnicity. Chronic renal failure was not considered as a confounder as it is on the causal pathway. Exposure to steroid medication was also not included as secondary diabetes Read codes were excluded. As age and gender were used in the matching process, we only included age in our final models to adjust for any residual confounding by this factor. BMI, alcohol intake and smoking status were taken from data recorded closest to index or matched index date. Ethnicity data was generated using both CPRD and HES data for English patients based on algorithms produced by one of the authors (RM) described previously [10]. See Additional file 3 for further methodology details on variable construction.

To explore any possible ascertainment bias from likely increased consultation rates in patients with diabetes, we studied the effect of diabetes within strata of yearly consultation rate and total consultation number. As consultation rates capture the severity of disease we did not adjust for this, to avoid adjusting for diabetes exposure. Yearly consultation rate was generated using the same face to face consultation codes used to define active patients and the denominator of follow-up time adjusted for any possible time-related bias from previous TB diagnoses. Similarly, each patient’s total consultation number was constructed for the same period.

We explored the effect of health care utilisation to investigate effects of access to care to reduce ascertainment bias for diabetes patients more likely to have regular primary care follow-up. Health care utilisation was captured by studying rates of blood pressure and cholesterol measurement and influenza vaccination. These three measures are incentivised in UK General Practice and patients with diabetes should receive these interventions at least once per year. These specific factors were chosen to capture health care utilisation without the same inherent limitation of consultation rate, which is dependent on diabetes severity.

### Statistical analysis

Follow-up time started from the index or matched-index or at the end of any potential immortal time period (Fig. 3). Follow-up ended at the earliest occurrence of first TB diagnosis post index date, transfer out of the practice, last practice data collection, death from any cause or the end of the study, 31 December 2012.

Initial univariate analysis of diabetes exposure and the outcome of TB were explored by dividing the exposed cohort into type 1 and type 2 diabetes categories to ascertain number of events in each and the possibility of further exploring these subtypes in the multivariable analysis.

**Fig. 4:**
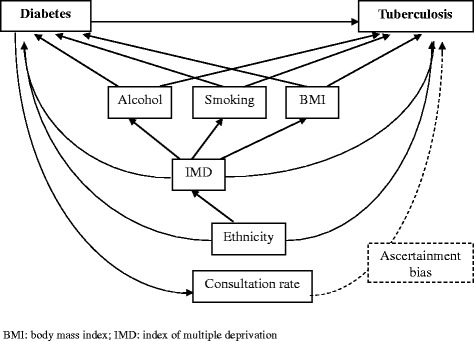
Causal diagram of associations between diabetes, tuberculosis and confounders

We used a Poisson regression model for the adjusted rate ratio of first diagnosis of TB comparing patients with diabetes with their matched unexposed cohort. We assumed that UK incidence rates of TB do not vary rapidly over time. An unmatched analysis was used as this does not introduce bias when analysing matched cohort studies [11]. The analysis model was developed guided by the postulated causal relationships between variables (Fig. 4). Variables were kept in the model if they changed the point estimate for the rate ratio of TB for our exposed cohort and with the addition of each variable we assessed for possible collinearity by studying changes in standard errors.

A priori effect modification of the relationship between diabetes and TB by age, ethnicity, duration of diabetes and within strata of consultation pattern was explored.

To explore the effect of diabetes exposure on absolute tuberculosis incidence we used the Poisson model developed previously to predict event rates amongst patients at most and least risk of TB according to our baseline bivariate analyses.

A pre-specified exploration of the effect of severity of diabetes was undertaken by comparing the adjusted rate ratios of TB incidence for patients with type 2 diabetes in different insulin treatment categories, comparing those requiring insulin and those without insulin (treated with other anti-diabetic drugs or diet alone) with a matched non-exposed cohort. A time-updated analysis was used (see Additional file 3 for a full description of the methodology).

The effect of glycaemic control was studied using a post-index mean value for HbA1c, calculated for all patients with type 2 diabetes and TB rates were calculated within strata of glycaemic control. Algorithms were developed to identify and convert HbA1c units from DCCT (Diabetes Control and Complications Trial) percent units to the more recent IFCC (International Federation of Clinical Chemistry) mmols/mol units.

All primary analyses were performed on a complete-case basis. Further post hoc and sensitivity analyses are described in Additional file 3. All data analyses were carried out using STATA 13 MP.

### Protocol and ethical approval

The study received ethical approval under protocol number 13_014 by the Independent Scientific Advisory Committee for the Medicines and Healthcare products Regulatory Agency for patient-level NHS database research and the LSHTM Research Ethics Committee (reference 6352).

## Results

### Study population and baseline characteristics

From 530,994 patients identified with a diabetes Read code recorded within the study period, 222,731 patients with incident diabetes were included in this study, comprising 6,186 patients with type 1 and 216,545 patients with type 2 diabetes (see Additional files 1 and 2). These were matched to a total of 1,218,616 patients who were unexposed to diabetes at their matched index date, 82,208 (6.74 %) of whom went on to have a diabetes Read code and hence joined the exposed cohort for the remainder of their follow-up. On initial matching 98.59 % of the exposed cohort had 6 possible matched controls with only 76 patients having no matched controls. After excluding these and controls that failed quality checks and were not active in their follow-up, 92.77 % of the exposed cohort had ≥5 matched controls.

The median follow-up time was 4.4 years (interquartile range 1.9 to 7.8 years) for patients with incident diabetes and 3.8 years (interquartile range 1.6 to 7.0 years) for matched control patients. In total, the diabetes cases and their matched controls contributed a little less than seven million person-years of follow-up. Amongst the cohort with diabetes, 66,005 (29.6 %) had no anti-diabetic medication recorded in their therapy files. In those patients with anti-diabetic prescriptions, 133,503 (85.2 %) had prescriptions for metformin singly or combined and 35,203 (22.4 %) had insulin prescriptions.

Baseline characteristics for patients with incident diabetes and the matched unexposed cohort are shown in Table 1. There were considerable missing data for ethnicity (45.3 %) and index of multiple deprivation (IMD) (39.5 %) for the total cohort, although there were fewer missing data for patients with diabetes. In total, 97,861 (43.9 %) patients with incident diabetes and 461,792 (37.9 %) of the matched unexposed cohort had complete data on all variables and were used in the complete case analysis. Patients with diabetes consulted more frequently than their matched controls (*P* <0.001).

**Table 1:** Characteristics of patients with incident diabetes and a matched^a^ non-exposed cohort

Characteristic	Patients with diabetes^b^	Patients without diabetes^c^
	Number = 222,731	Number = 1,218,616
Age at entry in years		
(Median, IQR)	62.9 (52.5–72.5)	63.6 (53.3–72.9)
Male gender		
(number, %)	122,594 (55.0)	647,287 (53.1)
Smoking (number, %)		
Non-smoker	82,856 (37.2)	525,881 (43.2)
Ex-smoker	93,491 (42.0)	404,929 (33.2)
Current smoker	42,414 (19.0)	244,247 (20.0)
Missing	3,970 (1.8)	43,559 (3.6)
Alcohol intake (number, %)		
Non-drinker	31,232 (14.0)	134,862 (11.1)
Ex-drinker	12,151 (5.5)	44,158 (3.6)
Moderate drinker	145,695 (65.4)	793,877 (65.2)
Heavy drinker	18,843 (8.5)	99,444 (8.2)
Missing	14,810 (6.7)	146,275 (12.0)
BMI (number, %)		
<20	4,648 (2.1)	50,404 (4.1)
20- < 25	29,973 (13.5)	347,150 (28.5)
25- < 30	70,949 (31.9)	418,421 (34.3)
≥30	105,789 (47.5)	242,928 (19.9)
Unknown	11,372 (5.1)	159,713 (13.1)
Ethnicity (number, %)		
White	122,507 (55.0)	626,733 (51.4)
South Asian	5,326 (2.4)	12,979 (1.1)
Black	2,347 (1.1)	7,979 (0.7)
Mixed or Other	2,143 (1.0)	8,518 (0.7)
Missing	90,408 (40.6)	562,407 (46.2)
Index of Multiple Deprivation		
1 – Least deprived	26,071 (11.7)	164,172 (13.5)
2	30,191 (13.6)	174,611 (14.3)
3	27,406 (12.3)	149,262 (12.3)
4	27,207 (12.2)	136,276 (11.2)
5 – Most deprived	24,080 (10.8)	113,147 (9.3)
Missing	87,776 (39.4)	481,148 (39.5)
Mean number of consultations per year		
1^st^ tertile^d^ (0–6)	16,096 (7.2)	464,352 (38.1)
2^nd^ tertile (7–12)	73,287 (32.9)	407,155 (33.41)
3^rd^ tertile (13-maximum)	133,348 (59.9)	347,109 (28.5)
Total number of consultations		
1^st^ tertile^d^ (1–15)	32,699 (14.7)	444,234 (36.5)
2^nd^ tertile (16–49)	65,760 (29.5)	411,258 (33.8)
3^rd^ tertile (50-maximum)	124,272 (55.8)	363,124 (29.8)
Length of follow-up in years		
(Median, IQR)	4.4 (1.9–7.8)	3.8 (1.6–7.0)
Total person-years	1,172,829	5,767,599

^a^Patients with and without diabetes were matched for index date, age +/− 5 years, gender and practice

^b^Diabetes diagnosis defined as index date and start of follow-up

^c^Patients without diabetes on matched index date but may become diabetic in follow-up period

^d^Tertiles defined in the overall cohort

*BMI* body mass index, *IQR* interquartile range

### Effect of diabetes on tuberculosis incidence

A total of 969 TB outcomes were recorded during follow-up and 2,376 (1.07 %) of the exposed and 13,867 (1.14 %) of the unexposed had previous TB recorded. The incidence of TB was higher amongst patients with diabetes compared with a matched unexposed group, 16.2 and 13.5 cases per 100,000 person-years, respectively (Table 2). After adjustment for potential confounders the association between diabetes and TB incidence remained (adjusted rate ratio 1.30, 95 % CI 1.01 to 1.67, *P* = 0.04).

**Table 2:** Rates and adjusted rate ratios for all types of tuberculosis (TB) by exposure to diabetes

		Age-adjusted		Fully adjusted model^a^
Exposure status	Number of TB cases/PY^b^	Rate^c^ (95 % CI)	Rate ratio	Rate ratio
			(95 % CI)	(95 % CI)
			P* = 0.025	P** = 0.039
Patients without diabetes	779/57.68	13.51 (12.59–14.49)	1.00	1.00
Patients with diabetes	190/11.73	16.20 (14.05–18.68)	1.20 (1.02–1.40)	1.30 (1.01–1.67)

*P-value from Wald test, **P-value from likelihood ratio test

^a^Model adjusted for: age, alcohol, smoking, BMI, ethnicity, IMD

^b^PY: 100,000 person years at risk

^c^Rate: per 100,000 person years

*BMI* body mass index, *CI* confidence interval, *IQR* interquartile range

The associations between diabetes and the potential confounders with TB that were included in the adjusted model are shown in Table 3.

**Table 3:** Univariate and multivariable associations between diabetes and other risk factors for tuberculosis

Variable	Crude rate ratio	Adjusted rate ratio^a^
	(95 % CI)	(95 % CI)
No diabetes	1.00	1.00
Diabetes	1.20 (1.02–1.41)	1.30 (1.01–1.66)
Age (per 1 year increase)	1.02 (1.01–1.03)	1.03 (1.02–1.04)
Smoking		
Non-smoker	1.00	1.00
Ex-smoker	1.23 (1.06–1.44)	1.27 (1.00–1.61)
Current smoker	1.73 (1.48–2.03)	1.67 (1.27–2.19)
Alcohol intake		
Non-drinker	1.00	1.00
Ex-drinker	0.64 (0.44–0.94)	1.00 (0.58–1.73)
Moderate drinker	0.50 (0.42–0.59)	0.86 (0.65–1.13)
Heavy drinker	0.66 (0.51–0.85)	1.04 (0.69–1.59)
BMI^b^		
<20	1.00	1.00
20- < 25	0.41 (0.33–0.51)	0.62 (0.42–0.90)
25- < 30	0.26 (0.21–0.32)	0.35 (0.24–0.51)
≥30	0.21 (0.17–0.27)	0.29 (0.19–0.45)
Ethnicity		
White	1.00	1.00
South Asian	9.41 (7.48–11.84)	10.77 (7.84–14.81)
Black	3.47 (2.11–5.72)	2.28 (1.01–5.16)
Mixed or Other	2.84 (1.70–4.75)	2.50 (1.23–5.07)
Index of Multiple Deprivation^c^		
1 – Least deprived	1.00	1.00
2	1.45 (1.10–1.91)	1.24 (0.89–1.72)
3	1.38 (1.03–1.84)	1.19 (0.85–1.67)
4	1.76 (1.33–2.33)	1.36 (0.97–1.90)
5 – Most deprived	2.31 (1.75–3.04)	1.47 (1.05–2.07)
Consultation rate^d^		
1^st^ tertile (0–6)	1.00	N/A
2^nd^ tertile (7–12)	2.08 (1.71–2.54)	
3^rd^ tertile (13-maximum)	4.58 (3.81–5.51)	
Total number of consultations^e^		
1^st^ tertile (1–15)	1.00	N/A
2^nd^ tertile (16–49)	0.54 (0.46–0.63)	
3^rd^ tertile (50-maximum)	0.26 (0.23–0.31)	

^a^Model includes: diabetes, age, smoking status, alcohol status, BMI, ethnicity and index of multiple deprivation

^b^BMI: body mass index in kg/m^2^

^c^Index of multiple deprivation in quintiles and available for English patients only

^d^Consultation rate defined as number of face to face consultations per year of follow-up; tertiles in order of increasing consultation rate

^e^Consultation number defined as total number of face to face consultations during patient follow-up; tertiles in order of increasing number of consultations

*CI* confidence interval, *NA* not available

There was only one TB outcome event in the follow-up period of patients classified with type 1 diabetes; thus, in all further analyses we used a cohort with both types of diabetes combined or where stated only those with type 2 diabetes and their matched unexposed cohort.

The estimated absolute increase in TB incidence comparing patients with and without diabetes in those with the highest baseline risk of TB (age >70 years, South Asian ethnicity, non-drinkers, current-smokers, BMI <20 and in the 5^th^ IMD quintile) was an extra 206 cases per 100,000 person-years (Table 4).

**Table 4:** Estimates^a^ of effect of diabetes on absolute tuberculosis incidence rates^b^

	Low risk group^c^			High risk group^d^		
	With diabetes	Without diabetes	Difference	With diabetes	Without diabetes	Difference
Tuberculosis rates^b^	3.7 × 10^−6^	2.8 × 10^−6^	8.4 × 10^−7^	893.7	687.9	205.8

^a^Incidence rates predicted from Poisson model including variables: diabetes status, age, ethnicity, alcohol status, smoking status, body mass index (BMI) and index of multiple deprivation (IMD)

^b^Tuberculosis rates per 100,000 person years

^c^Age <20 years, white ethnicity, moderate drinkers, non-smokers, BMI >30 and in the 1^st^ IMD quintile

^d^Age >70 years, South Asian ethnicity, non-drinkers, current-smokers, BMI <20 and in the 5^th^ IMD quintile

### Effect of age, duration of diabetes diagnosis and consultation patterns

The median age for TB diagnosis was nearly five years younger for patients with diabetes (67.5 years, IQR 58.5–75.4 years) compared with unexposed patients (71.9 years, IQR 63.5–78.3 years, *P* <0.001, Wilcoxon rank sum test). There was no evidence for an effect of age on diabetes risk for tuberculosis (*P* = 0.228), although there were only small numbers of patients in the lowest age strata (Table 5). There was no evidence for effect of duration of diabetes diagnosis on risk of tuberculosis (*P* = 0.375). To study possible interaction with ethnicity the categories of black and mixed/other were combined to provide sufficient TB events in each strata. There was no evidence for a role of ethnicity in modifying the effects of diabetes for tuberculosis risk (*P* = 0.894).

**Table 5:** Effect of diabetes (DM) on rate of tuberculosis, modification by consultation rate, consultation number, age, time since diagnosis of diabetes and ethnicity

Strata	Exposure status	TB cases/PY^a^	Unadjusted rate	Unadjusted rate ratio	Adjusted rate ratio^b^	*P*-value for interaction^c^
			(95 % CI)	(95 % CI)	(95 % CI)	
Consultation rate^d^						
1^st^ tertile	DM	5/0.93	5.35 (2.23–12.86)	0.91 (0.37–2.22)	1.80 (0.64–5.01)	
	Non-DM	141/23.94	5.89 (4.99–6.95)	1.0	1.0	
2^nd^ tertile	DM	39/4.61	8.46 (6.18–11.59)	0.65 (0.46–0.91)	1.01 (0.63–1.62)	0.535
	Non-DM	273/20.92	13.05 (11.59–14.69)	1.0	1.0	
3^rd^ tertile	DM	146/6.19	23.60 (20.01–27.76)	0.83 (0.68–1.00)	0.94 (0.69–1.27)	
	Non-DM	265/12.81	28.50 (25.72–31.57)	1.0	1.0	
Consultation number^e^						
1^st^ tertile	DM	48/0.35	138.41 (104.31–183.67)	5.14 (3.78–7.00)	2.99 (1.68–5.31)	
	Non-DM	254/9.43	26.93 (23.82–30.46)	1.0	1.0	
2^nd^ tertile	DM	70/1.97	35.61 (28.17–45.01)	2.42 (1.86–3.14)	2.88 (1.96–4.24)	0.003
	Non-DM	285/19.37	14.71 (13.10–16.53)	1.0	1.0	
3^rd^ tertile	DM	72/9.42	7.65 (6.07–9.63)	0.92 (0.71–1.20)	1.26 (0.87–1.83)	
	Non-DM	240/28.87	8.31 (7.32–9.43)	1.0	1.0	
Age in years						
0- < 45	DM	10/1.10	9.07 (4.88–16.86)	1.23 (0.61–2.45)	0.97 (0.32–2.93)	
	Non-DM	41/5.54	7.40 (5.45–10.04)	1.0	1.0	
45- < 70	DM	103/6.11	16.85 (13.89–20.45)	1.71 (1.37–2.14)	1.62 (1.14–2.31)	0.228
	Non-DM	295/29.93	9.86 (8.79–11.05)	1.0	1.0	
70+	DM	77/4.51	17.0 (13.64–21.32)	0.85 (0.67–1.09)	1.08 (0.76–1.55)	
	Non-DM	443/22.20	19.95 (18.18–21.90)	1.0	1.0	
Time since index date						
0- < 1 years	DM	56/2.07	27.07 (20.83–35.17)	1.83 (1.35–2.48)	1.48 (0.86–2.56)	
	Non-DM	165/11.16	14.78 (12.69–17.22)	1.0	1.0	
1- < 5 years	DM	80/5.72	13.99 (11.24–17.41)	0.99 (0.78–1.26)	1.24 (0.88–1.74)	0.375
	Non-DM	407/28.81	14.13 (12.82–15.57)	1.0	1.0	
5- < 10 years	DM	41/3.08	13.32 (9.80–18.08)	1.05 (0.75–1.48)	1.13 (0.68–1.87)	
	Non-DM	177/14.01	12.64 (10.91–14.64)	1.0	1.0	
10+ years	DM	13/0.86	15.11 (8.77–26.02)	1.86 (0.97–3.57)	2.66 (1.12–6.35)	
	Non-DM	30/3.70	8.11 (5.67–11.61)	1.0	1.0	
Ethnicity						
White	DM	74/6.74	10.98 (8.74–13.79)	0.88 (0.69–1.13)	1.27 (0.95–1.69)	
	Non-DM	380/30.57	12.43 (11.24–13.75)	1.0	1.0	
South Asian	DM	38/0.27	139.40 (101.43–191.58)	1.39 (0.91–2.12)	1.34 (0.79–2.28)	0.894
	Non-DM	49/0.49	100.58 (76.02–133.08)	1.0	1.0	
Black, Mixed or Other	DM	5/0.20	24.41 (10.16–58.65)	0.57 (0.22–1.49)	1.65 (0.55–4.93)	
	Non-DM	26/0.61	42.74 (29.10–62.77)	1.0	1.0	

^a^PY: 100,000 person years at risk

^b^Model adjusted for: age, ethnicity, BMI, alcohol status, smoking and IMD

^c^ *P-*value from likelihood ratio test

^d^Consultation rate defined as number of face to face consultations per year of follow-up; tertiles in order of increasing consultation rate

^e^Consultation number defined as total number of face to face consultations during patient follow-up; tertiles in order of increasing number of consultations

*BMI* body mass index, *CI* confidence interval, *IMD* index of multiple deprivation, *TB* tuberculosis

For consulting patterns, there was a suggestion that diabetes had a greater effect on risk of TB for those who consulted at the lowest rate and the effect of diabetes diminished with increasing consultation rates, but this pattern was not statistically significant. Our results were not changed by excluding those with follow-up shorter than six months who might have unstable consultation rates. A similar but statistically significant pattern was seen when looking within strata of total consultation number with the effect of diabetes conferring a greater risk for TB in the group with the least number of consultations in their follow-up (*P* = 0.003).

Across health care utilisation patterns there was strong evidence (*P* <0.001) of differences in TB risk amongst diabetes patients, with patients accessing the lowest and highest amounts of chronic disease health care being at higher risk of TB, particularly in those with lower influenza vaccination rates (Table 6).

**Table 6:** Comparison of rates of tuberculosis in 216,545 patients with type 2 diabetes (T2DM) categorised by health care utilisation with a matched unexposed cohort

			Age adjusted		Fully adjusted model^a^	
Exposure group	Number of patients	TB cases/PY^b^	Rate^c^ (95 % CI)	Rate ratio	Rate ratio	*P*-value*
	(% of T2DM)			(95 % CI)	(95 % CI)	
Patients without diabetes	1,186,844	764/55.87	13.67 (12.74–14.68)	1.00	1.00	
Patients with T2DM						
Cholesterol testing rate:						
Less than once per year	89,989 (41.6)	116/4.42	26.23 (21.87–31.46)	1.91 (1.58–2.33)	1.78 (1.29–2.44)	<0.001
Once per year	88,961 (41.1)	44/5.52	7.97 (5.93–10.71)	0.58 (0.43–0.79)	0.76 (0.50–1.14)	
Twice or more per year	37,595 (17.3)	29/1.44	20.16 (14.01–29.01)	1.47 (1.02–2.14)	1.98 (1.20–3.27)	
Blood pressure testing rate:						
Up to once per year	41,839 (19.3)	55/1.94	28.30 (21.73–36.86)	2.34 (1.78–3.07)	2.49 (1.59–3.92)	<0.001
Once to twice per year	55,983 (25.9)	27/3.36	8.03 (5.51–11.72)	0.60 (0.41–0.88)	0.67 (0.38–1.18)	
More than twice per year	118,723 (54.8)	108/6.42	16.81 (13.92–20.30)	1.23 (1.00–1.50)	1.34 (0.99–1.81)	
Influenza vaccination rate:						
Less than once per year	57,257 (26.4)	84/2.35	35.62 (28.76–44.11)	2.61 (2.08–3.26)	2.91 (2.00–4.23)	<0.001
Once per year	159,288 (73.6)	105/9.02	11.64 (9.61–14.09)	0.85 (0.69–1.04)	0.98 (0.73–1.32)	

*P-value from likelihood ratio test

^a^Model adjusted for: age, BMI, smoking status, alcohol status, ethnicity and IMD

^b^PY: 100,000 person years at risk

^c^Rate: per 100,000 person years

*BMI* body mass index, CI confidence interval, *IMD* index of multiple deprivation, *TB* tuberculosis

### Comparing insulin and non-insulin users within type 2 diabetes

There was an association between increasing severity of type 2 diabetes and increasing TB risk as indicated by time-dependent analyses comparing insulin users versus non-users in crude analyses with age adjustment (*P* = 0.020), but this relationship did not remain after full adjustment (Table 7).

**Table 7:** Comparing rates of tuberculosis in 216,545 patients with type 2 diabetes (T2DM) by time-dependent treatment group with a matched unexposed cohort

			Crude		Fully adjusted model^a^	
Exposure group	Number of patients	TB cases/PY^b^	Rate^c^ (95 % CI)	Rate ratio	Rate ratio	*P*-value*
	(% of T2DM)			(95 % CI)	(95 % CI)	
Patients without diabetes	1,186,932	765/55.87	13.69 (12.76–14.70)	1.00	1.00	
Patients with T2DM:						
Without insulin exposure	190,865 (88.1)	161/10.05	16.02 (13.72–18.69)	1.16 (0.98–1.37)	1.27 (0.98–1.66)	0.119
During insulin exposure	25,680 (11.9)	28/1.33	21.07 (14.55–30.51)	1.63 (1.12–2.38)	1.49 (0.83–2.67)	

*P-value from likelihood ratio test

^**a**^Model adjusted for: age, BMI, smoking status, alcohol status, ethnicity and IMD

^**b**^PY: 100,000 person years at risk

^**c**^ Rate: per 100,000 person years

*BMI* body mass index, CI confidence interval, *IMD* index of multiple deprivation, *TB* tuberculosis

### Effect of glycaemic control in type 2 diabetes

In the subset of type 2 diabetes patients with Hba1c measurements (198,227) compared with their matched unexposed controls, there was no evidence for increasing TB risk with worsening glycaemic control (Table 8).

**Table 8:** Comparing rates of tuberculosis in 198,227 patients with type 2 diabetes (T2DM) and HbA1c measurements with a matched unexposed cohort

			Age adjusted		Fully adjusted model^a^	
Exposure group	Number of patients	TB cases/PY^b^	Rate^c^ (95 % CI)	Rate ratio	Rate ratio	*P*-value*
	(% of T2DM)			(95 % CI)	(95 % CI)	
Patients without diabetes	1,088,376	689/50.97	13.51 (12.54–14.56)	1.00	1.00	
Patients with T2DM						
HbA1c mmol/mol (%):						
≤48 (6.5)	61,336 (30.9)	50/2.86	17.48 (13.25–23.06)	1.29 (0.97–1.72)	1.34 (0.88–2.05)	0.140
49- ≤ 58 (6.5–7.5)	70,470 (35.6)	54/3.98	13.57 (10.39–17.72)	1.00 (0.76–1.32)	1.14 (0.76–1.70)	
>58 (>7.5)	66,421 (33.5)	71/3.99	17.81 (14.12–22.48)	1.32 (1.03–1.68)	1.50 (1.04–2.17)	

*P-value from likelihood ratio test

^**a**^Model adjusted for: age, BMI, smoking status, alcohol status, ethnicity and IMD

^**b**^PY: 100,000 person years at risk

^**c**^Rate: per 100,000 person years

*BMI* body mass index, CI confidence interval, *IMD* index of multiple deprivation, *TB* tuberculosis

Post hoc and sensitivity analyses are described in Additional file 4.

## Discussion

### Key findings

In this large UK population based cohort of patients with incident diabetes we found only an overall modest 1.3 fold increased risk of tuberculosis. We found no evidence for higher relative increases in TB rates amongst diabetes patients of different age groups or ethnicities, longer duration of disease, those using insulin or with worse glycaemic control. There was strong evidence for differences amongst diabetes patients with different health care utilisation patterns. The highest risk of TB disease was amongst the group least accessing chronic disease health care.

### Comparison with other studies and explanation of findings

Our study is the largest cohort study to date exploring the association between diabetes and TB with individual-level adjustment for important demographic and lifestyle factors. The finding of an overall increased risk of TB in those with diabetes is in agreement with previous published studies and reviews [12–29, 4, 30, 3, 31]. However, in contrast we find only an overall small relative effect from diabetes in our UK population. The most recent systematic review and meta-analysis [3] included eight case-control studies with odds ratios ranging from 1.16 to 7.83 and a random effects analysis of the three included cohort studies showed a three-fold increased risk of TB with diabetes (relative risk 3.11, 95 % CI 2.27 to 4.26). All three cohort studies included in the meta-analysis were conducted in high TB incidence countries and two used cohorts of renal transplant patients.

Since the most recent systematic review, we are aware of seven more published analytical studies in humans looking at the question of TB risk associated with diabetes, including two case-control [26, 27] and five cohort studies [25, 28, 29, 4, 32] summarised in Additional file 5. Our finding of only a modest increase in risk of TB with diabetes is in agreement with these more recent studies. As study size increases there is a decrease in the estimate for the association between diabetes and TB seen for both cohort and case-control designs, even in higher incidence countries. These differences could be due to publication bias in earlier studies and/or the adjustment for more confounding in later studies. Leung et al. [25] used a cohort from a Hong Kong community based health program for ≥65 year olds and were able to adjust for demographic and lifestyle factors giving an overall adjusted hazard ratio of 1.77 (1.41 to 2.24). Dobler et al. [29] used a whole population cohort for Australian citizens and adjusted for demographic and indigenous status and TB incidence in country of birth using census aggregate data for the unexposed general population cohort. They found a 1.4 fold increased risk of TB in those with diabetes (RR 1.48, 1.04 to 2.10). The largest matched case-control study by Leegaard et al. [27] in Denmark found no overall association between diabetes and TB after adjusting for a range of chronic disease and demographic indicators (OR 1.18, 0.96 to 1.45).

We report a median length of follow-up of 4.4 years which is similar to that in previous studies and on the whole, most of the patients included had reasonably well-controlled diabetes. The largest cohort studies previously reported, Kim et al. [12] and Dobler et al. [29], which were unable to adjust for individual confounding, had study periods of two and six years, respectively. It is possible that the risk of TB associated with well-controlled diabetes only becomes manifest over much longer periods and we may possibly have under-estimated long-term risks.

The relatively small effect estimate for TB risk from diabetes found in our study could be due to the ability to adequately control for important individual level confounding from lifestyle and demographic risk factors using the CPRD. Our findings might also be a reflection of a successful primary care service with good chronic disease management and, therefore, reduction in attendant infection complications from diabetes. In support of this hypothesis, we see increased risks for TB disease from diabetes in those with the highest and lowest chronic disease management. Diabetes patients most frequently accessing chronic disease care could represent a group with more uncontrolled disease where general practice teams are seeking to improve diabetes management. The group with the least health care utilisation from that incentivised in UK General Practice for chronic disease management, may have more uncontrolled diabetes and have limited access to primary health care. The latter group might form part of a wider hard to reach group who are at increased risk of TB not only from diabetes but from multiple social risk factors [33]. Of note, our findings show diabetes patients receiving standard rates of chronic disease care, which in part will reflect good diabetes control, are at no increased risk of TB compared against patients without diabetes.

We did not find evidence for an effect of age, duration of diabetes or ethnicity for TB risk in diabetes patients although this could be due to type 2 error as we only had very small numbers of TB cases in some diabetes subgroups and had missing data on important confounders. Although some studies have found evidence for increasing TB risk in younger diabetes patients [12, 15], other authors have not [27, 29]. No previous studies have explored the effect of duration of diabetes diagnosis on risk of TB disease but if diabetes acts to increase the risk of TB infection or increases reactivation of latent disease, it would seem probable that cumulative exposure to diabetes would potentiate these risks.

The previous literature, using a variety of different markers, show mixed results for the effect of diabetes severity on the risk for TB. Leung et al. [25] stratified by glycaemic status and found those with Hba1c <7 % had no increased risk of TB compared with those without diabetes, in contrast to the subjects with Hba1c ≥7 % who were at 2.5 fold risk (HR 2.56, 1.95 to 3.35). Baker et al. [28] used the number of diabetes complications to explore the effect of diabetes severity and found that those with treated diabetes and ≥2 complications compared against a group without diabetes had a greater risk of TB (RR 3.45, 1.59 to 7.90). Dobler et al. [29] explored the effects of insulin use as a marker of severity and found that those using insulin had 2.3-fold risk compared against a general population cohort (RR 2.27, 1.41 to 3.66). In contrast, and coherent with our own data, Leegaard et al. [27] found no evidence for an association between TB risk and dysglycaemia. Again, this might reflect that diabetes patients managed in UK Primary Care have very well controlled disease, not completely captured by mean Hba1c measurements and, therefore, reduced attendant risks from infection.

We aimed to compare the different types of diabetes for risk of TB. The underlying hypothesis being that type 1 diabetes represents a more severe form of diabetes and thus we might expect that it poses a greater risk for TB infection if the relationship between diabetes and TB risk is causal. Only the previous study by Leegaard et al [27] defined and explored the risk of TB for a group with type 1 diabetes. They classified patients <30-years old using insulin monotherapy and never using oral antidiabetes medications as having type 1 diabetes. Our classification differed in that we defined our type 1 cohort using incentivised diagnostic codes and additional demographic factors to age and insulin prescriptions. Leegaard et al had very small numbers of patients classified with type 1 diabetes, only three amongst their TB cases and the adjusted TB risk estimate reflected the imprecision (OR 2.59, 0.44–15.29). Similarly, we found only one case of TB amongst our group of patients classified with type 1 diabetes and, thus, we were unable to explore the effects of type 1 diabetes further. Under-ascertainment of TB in type 1 diabetes patients within CPRD is a possible cause of our finding only one case of TB in this group. This might be due to those with type 1 diabetes mainly receiving their care in hospital out-patients clinics and notification of TB diagnoses not being returned to general practice. If a large number of cases of TB in the UK are due to reactivation of latent disease from those born in high TB burden countries [2, 1], it might be that incidence of type 1 diabetes in these populations is low, as supported by global incidence studies [34] or that these patients suffer competing risks before possible reactivation of TB infection.

Current UK guidelines advise considering treatment for latent TB infection in certain groups of adults where active disease has been ruled out but they show signs of TB infection with Mantoux positivity (≥6 mm) and without prior Bacillus Calmette-Guérin (BCG) vaccination, or strong Mantoux positivity (≥15 mm) or interferon-gamma release assay (IGRA) positive and with prior BCG vaccination [35]. There is no specific guidance for patients with diabetes at present.

### Strengths and limitations

To our knowledge, this is the largest cohort study to date exploring the association between diabetes and TB in a general population which was able to adjust for important individual level confounding demographic, socioeconomic and lifestyle factors. By using time-updated exposure status, where previous unexposed patients could later develop incident diabetes and join the exposed cohort, we could study time-related phenomena but our design also allowed comparison between more similar groups ensuring we could explore the role of diabetes with reduced confounding from unmeasured social and health-related risk factors. Previous literature describe excluding people with prior diagnoses of tuberculosis but we included this group with a suitable time-lapse as they will be amongst the highest risk groups for TB disease in the UK so our findings are more applicable to the population of interest.

Our study explores effects of important patient characteristics such as age and ethnicity and aspects of the risk factor of diabetes such as duration and severity, which have not been previously explored within one cohort. As far as we are aware this is the only study to look at how TB risk for diabetes patients varies with consultation patterns including receipt of chronic disease health care and shows how UK General Practice systems are able to identify different risk groups. The study is based within the General Practice population using routinely collected clinical data so reducing the likelihood of significant selection bias. In the UK the majority of diabetes care occurs in the primary care setting making our cohort of diabetes patients very inclusive. Where patients receive specialist diabetes care in the face of more challenging disease control, their primary care record is equally important and contemporaneous as primary care co-ordinates ongoing management, such as retinal screening and immunisation. Our study is pertinent to any UK policy seeking to identify high-risk groups in primary care suitable for latent TB infection screening using such routine electronic health data.

There is the potential for misclassification of diabetes and TB within our study as we used routinely collected clinical data without validation from consultation free text or hospital correspondence. We expect misclassification to be minimal as diabetes is an indicator condition within UK Primary Care; thus, practices are incentivised to maintain accurate diabetes patient registers. Seventy percent of our cohort with diabetes had confirmatory prescription data for anti-diabetic medications and the 30 % having no specific therapy but other recorded indicators is in agreement with previous studies [36]. We generated a specific TB Read code list to try to avoid the inclusion of non-mycobacterial disease. Although TB can present non-specifically initially we would expect patients to seek medical attention in a setting with free access to health care. Diagnoses of TB made in secondary care are highly likely to be communicated to the General Practitioner due to the public health risk of this communicable disease, the risk of serious side-effects from antituberculous therapy and possible treatment interactions with medications prescribed in primary care. We found an incidence of TB in our unexposed cohort that was equal to that reported as the UK TB incidence for 2012 (13.9 cases per 100,000 population) [2], therefore supporting that we reliably identified cases of TB. Any misclassification of our outcome is likely to be non-differential as unexposed patients could join the exposed group during follow-up and “inactive” controls were excluded who are more likely to be misclassified as unexposed. Furthermore, there is no current UK guideline advising screening of patients with diabetes for TB and broader awareness for the association between the two conditions was only recently raised [37], thus any bias will tend towards underestimating associations.

There is a risk of reverse causality with diagnoses made close together and with a condition such as TB which may initially present with non-specific symptoms and can itself cause a reactive hyperglycaemia associated with acute infection [38]. TB diagnosis and treatment delays can be as long as three months in high-income settings [39, 40] but potential delays in diagnosis of diabetes far exceed this with estimates between four to seven years [41]. Thus, it is far more likely in the situation where a diagnosis of TB closely follows a diagnosis of diabetes that the condition of diabetes or chronic hyperglycaemia has been ongoing for some time previous. Using similar considerations, we did not introduce a minimum follow-up time after diabetes diagnosis. There is no current evidence-base to guide us in considering a mechanistically plausible minimum length of follow-up within which TB risk increases and introducing an arbitrary follow-up time will only produce survivor bias.

Information on country of origin is not routinely recorded within the primary care clinical record and may confound the association between diabetes and TB. Lifestyle factors predisposing a person to diabetes associated with the country of origin are likely superseded by those associated with residence in the UK and remaining lifestyle factors or genetic predisposition could be crudely captured by ethnicity descriptors. However, there is likely residual confounding especially as ethnicity was only broadly described within our study. We did not identify and adjust for HIV status within our study as this is recognised to be under-reported in General Practice records [42]. HIV and its treatment have been associated with metabolic derangement including diabetes [43] and HIV is a strong risk factor for TB disease [44, 45]. We did not identify and exclude patients with recorded HIV as this may have differentially affected TB patients with diabetes as we expected these patients might be more likely to share a diagnosis of HIV with their General Practitioner due to the risk of multiple drug interactions between their HIV and diabetes medications. We did not adjust for BCG vaccination status. The UK immunisation program recommends childhood BCG immunisation for those at higher risk of TB [46]. The protection offered by BCG immunisation wanes with time [47], and is likely to offer little protection to the age groups predominantly at risk of TB associated with diabetes.

We performed multiple imputation for missing data on important covariates but there is likely residual confounding from non-differential misclassification of these data. Our main findings were robust to the inclusion of imputed missing data. There were fewer missing data for patients with diabetes, which is to be expected as these represent a group that are better characterised within routine primary care data. Missing data produce loss of study power, despite multiple imputation methods, which calculate standard errors reflecting the increased uncertainty. The study also had insufficient power, risking type 2 error, for the investigation of some interactions as we had small numbers of outcome data in some of our subgroup analyses. Thus, larger well-characterised cohorts in higher TB incidence settings would be needed to study these possible interactions.

## Conclusions and public health implications

We find evidence for an increased risk of TB amongst those with diabetes but in contrast with many other studies mostly from high TB burden countries, the risk is only modest in our UK General Practice population. Our findings may reflect good management of diabetes in UK primary care and, therefore, reduced attendant infection risks from this chronic condition. Diabetes alone is not a sufficient indication for screening for latent TB infection in the UK. Importantly we also identified a group of patients with diabetes and reduced primary health care utilisation who are at increased risk of TB. It appears these might form a higher risk hard to reach group in terms of accessing them through primary care. Our findings can inform the development of such a primary care latent TB infection screening program hoping to reach high-risk groups and how effective such an approach might be for improving UK TB control.

## Additional files

Additional file 1:
**Algorithm for categorising patients with type 1 diabetes.** OAD: other anti-diabetic drugs (not metformin or insulin). ^1^Younger age cut-off for high-risk ethnicities: black, South Asian, other or mixed. Additional file 2:
**Algorithm for categorising patients with type 2 diabetes.** OAD: other anti-diabetic drugs (not metformin or insulin). ^1^Continuous insulin defined as no gap between prescriptions >6 months and prescription in the last 12 months. ^2^Younger age cut-off for high-risk ethnicities: black, South Asian, other or mixed. Additional file 3:
**Methodology.**
Additional file 4:
**Results of sensitivity and post-hoc analysis.**
Additional file 5:
**Summary of observational studies published since 2008 investigating the association between diabetes (DM) and tuberculosis (TB) risk.** ^1^BMI: body mass index, ^2^ CVD: cardiovascular disease, ^3^HTN: hypertension, ^4^COPD: chronic obstructive pulmonary disease, ^5^ADL: activities of daily living and ^6^ICD-9-CM: International Classification of Disease, Ninth Revision, Clinical Modification.
