# Supplementary figures and images for: Risk of tuberculosis in patients with diabetes: population based cohort study using the UK Clinical Practice Research Datalink
Source: BMC Med. 2015 Jun 5;13:135. doi: 10.1186/s12916-015-0381-9 (PMC4470065; doi:10.1186/s12916-015-0381-9)

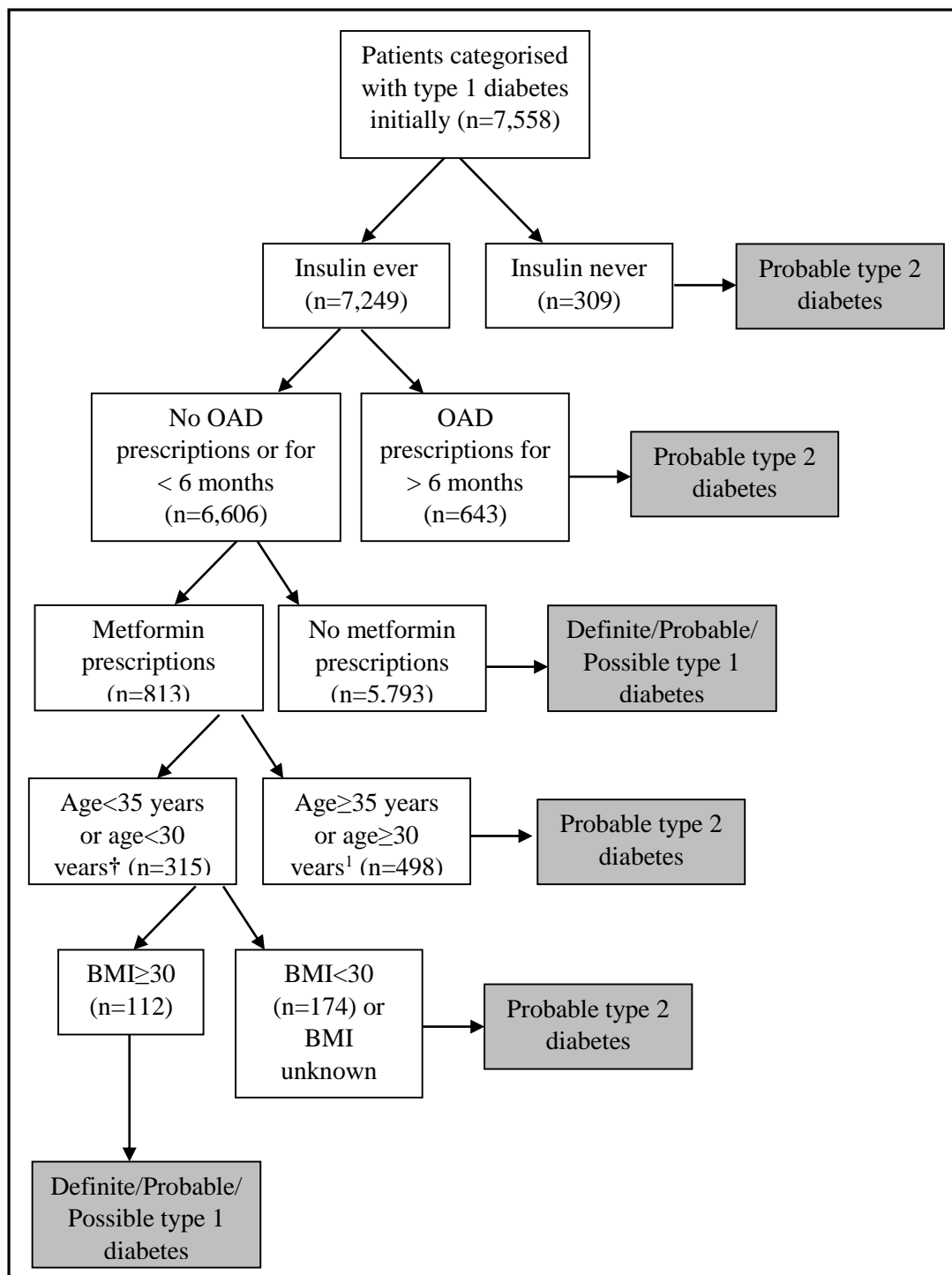

Supplement: Additional file 1: — Algorithm for categorising patients with type 1 diabetes. OAD: other anti-diabetic drugs (not metformin or insulin). 1Younger age cut-off for high-risk ethnicities: black, South Asian, other or mixed. [file 12916_2015_381_MOESM1_ESM.pdf]

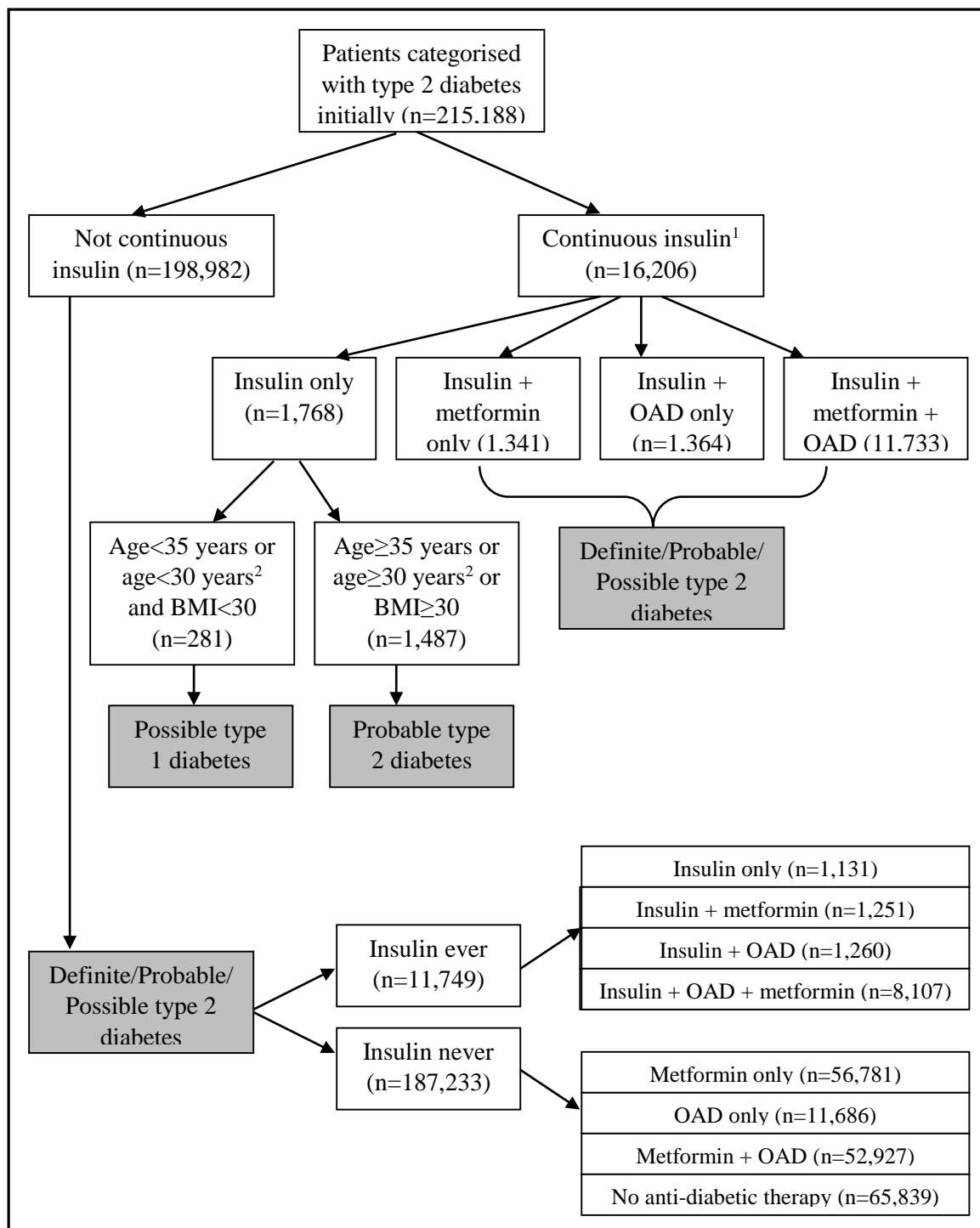

Supplement: Additional file 2: — Algorithm for categorising patients with type 2 diabetes. OAD: other anti-diabetic drugs (not metformin or insulin). 1Continuous insulin defined as no gap between prescriptions >6 months and prescription in the last 12 months. 2Younger age cut-off for high-risk ethnicities: black, South Asian, other or mixed. [file 12916_2015_381_MOESM2_ESM.pdf]
