# Supplementary material for: Risk of tuberculosis in patients with diabetes: population based cohort study using the UK Clinical Practice Research Datalink
Source: BMC Med. 2015 Jun 5;13:135. doi: 10.1186/s12916-015-0381-9 (PMC4470065; doi:10.1186/s12916-015-0381-9)
Supplement: Additional file 3: — Methodology. [file 12916_2015_381_MOESM3_ESM.docx]

## Additional File A3: Methodology

### Creating the cohort of patients with diabetes

We created our Read code list by searching the CPRD Read code dictionary using suitable search terms and then cross-referencing this with Quality Outcome Framework (QOF) diabetes code lists. QOF is the voluntary system introduced into UK general practice in 2004 to financially incentivise high quality care and includes practices maintaining chronic disease registers using specific diagnostic Read codes. In this way we created an inclusive list of diabetes Read codes (available on request). A hierarchical approach was taken describing codes as *definite, probable* or *possible* type 1 and 2 diabetes, depending on whether these diabetes types were referred to in the code text or were specific diabetes QOF codes.

The incident date for diabetes was taken as the first diabetes diagnostic Read code as opposed to earliest of anti-diabetic therapy prescription or diagnostic Read code as the use of some anti-diabetic medications is not specific to type 1 or 2 diabetes, particularly the first-line oral medication metformin[1]. We found that only 5.4% of our cohort identified with incident diabetes had prescriptions for possible anti-diabetic medications before their first diagnostic Read code, and for the majority of patients, these two dates were within two months of each other (median 49, interquartile range 9 to 447 days).

### Construction of confounder and effect modifier variables

BMI constructed from height and weight data was checked against feasible BMI ranges separately for children (5-17 years) and adults (≥18 years)[2]. Patient-level socioeconomic data were provided by CPRD in the form of quintile of index of multiple deprivation score (IMD) for English patients only that had this data. Ethnicity data was generated using both CPRD and HES data for English patients based on algorithms produced by one of the authors (RM) described previously[3]. Where ethnicity was missing from CPRD then HES data were used where available. When ethnicity codes were contradictory between the two databases then the most prevalent or latest (if joint prevalence) ethnicity descriptor was taken. Ethnicity was broadly divided into four main categories: black, white, south asian and mixed or other. Smoking status (non-, ex- and current smoker) and alcohol consumption (non-, ex-, moderate and heavy (>6 units per day) drinker) were defined using recorded status nearest to index date.

### Comparing patients with type 2 diabetes in different insulin treatment groups with a group without diabetes

A pre-specified exploration of the effect of severity of diabetes was undertaken by comparing the adjusted rate ratios of TB incidence for patients with type 2 diabetes in different insulin treatment categories, comparing those requiring insulin and those without insulin (treated with other antidiabetic drugs or diet alone) with a matched non-exposed cohort. A time-updated analysis with Lexis expansion was used. Patients with type 2 diabetes with >1 prescription for insulin were considered exposed to insulin from their first prescription of insulin until a TB outcome or until they were censored for any other reason. This was a pragmatic approach as 89% of the group with >1 prescription for insulin had a prescription within a year of the end of their follow-up, and due to the progressive nature of diabetes, it is likely these repeat prescriptions were for >1 month of treatment. For patients with type 2 diabetes and only one recorded prescription for insulin the end of insulin exposure was considered to be 30 days after their prescription date assuming that first prescriptions for insulin would be for one month equivalent of medication before they would need a clinical review. The period prior to insulin prescriptions was part of the non-insulin exposure time. In this way, the cohort of patients with type 2 diabetes was divided with follow-up time distributed between insulin and non-insulin exposure periods and compared against the subset of unexposed matched patients. These two exposure groups were studied in the same multivariable Poisson model described for the main analysis and we explored evidence against a linear dose-response with insulin exposure.

### Post hoc and sensitivity analyses

Post hoc analyses included exploration of the effect of calendar period broadly split into two periods of 1990-2000 and 2000-2012. We performed a post hoc sensitivity analysis restricting to the *definite* and *probable* cases of diabetes and their unexposed matched cohort to explore the effect of possible misclassification bias in exposure. Analyses were repeated excluding patients with TB events recorded previous to their index date.

We repeated our analyses including patients who had initially been excluded in the multivariable model due to missing values for ethnicity, socioeconomic and lifestyle covariates by assuming data were missing at random and using multiple imputation with the chained equations method[4]. Our imputation models were compatible with the substantive model and included the outcome, follow-up time represented by a cumulative baseline hazard and the auxiliary variables of gender and region of general practice[5]. We inspected distributions of imputed variables comparing against the observed data. We used 40 imputations to provide estimates that gave Monte-Carlo errors < 10% of standard errors.

### References

1. Solberg LI, Engebretson KI, Sperl-Hillen JM, Hroscikoski MC, O'Connor PJ. Are Claims Data Accurate Enough to Identify Patients for Performance Measures or Quality Improvement? The Case of Diabetes, Heart Disease, and Depression. American Journal of Medical Quality. 2006;21(4):238-45. doi:10.1177/1062860606288243.

2. Centres for Disease Control and Prevention. Growthcharts. <http://www.cdc.gov/growthcharts/percentile_data_files.htm>. Accessed 3 September 2013.

3. Mathur R, Bhaskaran K, Chaturvedi N, Leon DA, Vanstaa T, Grundy E et al. Completeness and usability of ethnicity data in UK-based primary care and hospital databases. Journal of public health (Oxford, England). 2013. doi:10.1093/pubmed/fdt116.

4. White IR, Royston P, Wood AM. Multiple imputation using chained equations: Issues and guidance for practice. Statistics in medicine. 2011;30(4):377-99. doi:10.1002/sim.4067.

5. Seaman SR, Bartlett JW, White IR. Multiple imputation of missing covariates with non-linear effects and interactions: an evaluation of statistical methods. BMC medical research methodology. 2012;12:46. doi:10.1186/1471-2288-12-46.
