# Supplementary material for: Risk of tuberculosis in patients with diabetes: population based cohort study using the UK Clinical Practice Research Datalink
Source: BMC Med. 2015 Jun 5;13:135. doi: 10.1186/s12916-015-0381-9 (PMC4470065; doi:10.1186/s12916-015-0381-9)
Supplement: Additional file 4: — Results of sensitivity and post-hoc analysis. [file 12916_2015_381_MOESM4_ESM.docx]

## Additional File A4: Results

### Post hoc and sensitivity analyses

There was no evidence for any effect of calendar period on risk of TB in diabetes (P-value= 0.794 for interaction, analyses on request). Limiting the analysis to include only a cohort of *definite* and *probable* cases of diabetes compared with their unexposed matched controls, did not have an effect on the patterns of association (adjusted RR 1.27, 0.97 to 1.67, P=0.087).

Excluding patients with diabetes and unexposed controls that had had previous TB recorded prior to index date did not change the pattern of association but effect estimates did not now reach significance (adjusted RR 1.22, 0.94 to 1.60, P=0.140).

Our study findings were robust to the inclusion of missing data for lifestyle, ethnicity and socioeconomic variables. Estimates were similar to our complete case analysis (adjusted RR 1.22, 1.04 to 1.45, P=0.018).
