# Supplementary material for: Risk of tuberculosis in patients with diabetes: population based cohort study using the UK Clinical Practice Research Datalink
Source: BMC Med. 2015 Jun 5;13:135. doi: 10.1186/s12916-015-0381-9 (PMC4470065; doi:10.1186/s12916-015-0381-9)
Supplement: Additional file 5: — Summary of observational studies published since 2008 investigating the association between diabetes (DM) and tuberculosis (TB) risk. 1BMI: body mass index, 2 CVD: cardiovascular disease, 3HTN: hypertension, 4COPD: chronic obstructive pulmonary disease, 5ADL: activities of daily living and 6ICD-9-CM: International Classification of Disease, Ninth Revision, Clinical Modification. [file 12916_2015_381_MOESM5_ESM.docx]

| **Table A5: Summary of the studies published since 2008 investigating the association between diabetes (DM) and tuberculosis (TB) risk using a cohort or case-control methodology** | | | | | | | | |
| --- | --- | --- | --- | --- | --- | --- | --- | --- |
| **Study** | **Country** | **Population** | **Study period** | **Exposure** | **Outcome** | **Confounders adjusted for** | **RR/OR**  **(95% CI)** | **Additional analyses** |
| **Cohort** |  |  |  |  |  |  |  |  |
| **Leung et al, 2008** | Hong Kong | Community-based health maintenance program for ≥65 year olds  477 TB cases  42,116 total cohort | 2000-2005 | DM diagnosed at baseline by fasting plasma glucose≥7.0 mmol/litre and/or blood/plasma glucose determinants | TB notification registry | Age, sex, smoking, alcohol, language, marital status, education, housing, working status, financial assistance status, BMI^1^, CVD^2^, HTN^3^, COPD^4^, asthma, malignancy, weight loss, hospital admission, ADL^5^ score | Active TB:  1.77 (1.41-2.24)  Culture confirmed:  1.91(1.45-2.52)  Pulmonary:  1.89 (1.48-2.42)  Extrapulmonary:  1.00 (0.54-1.86) | vs no DM:  DM Hba1c<7% - 0.81 (0.44-1.48)  Hba1c≥7% - 2.56 (1.95-3.35) |
| **Baker et al, 2012** | Taiwan | National Health Interview Survey linked with Taiwan National Health Insurance and death registry including ages≥12 years.  57 TB cases  17,715 total | 2001-2004 | DM diagnosed at baseline from self-report, ≥2 outpatient ICD-9-CM DM codes, ≥1 inpatient ICD-9-CM DM code or DM medication | Presence of all of ≥1 ICD-9-CM TB code and a prescription for ≥2 anti-TB drugs and no later misdiagnosis codes | Age, sex, crowding, smoking, household income, marital status, education, alcohol use, residence in an indigenous community | DM: 2.09 (1.10-3.95)  Treated DM: 2.60 (1.34-5.03) | DM severity from number of reported complications and ICD-9-CM^6^ component of Diabetes Complications Severity Index (DCSI).  vs no DM: |
|  | | | | | | | | |
|  | | | | | | | | |
| **Table A5 continued** | | | | | | | | |
| **Study** | **Country** | **Population** | **Study period** | **Exposure** | **Outcome** | **Confounders adjusted for** | **RR/OR**  **(95% CI)** | **Additional analyses** |
| **Baker et al continued** |  |  |  | Further divided as “DM” and “treated DM” |  | Receipt of government subsidy, employment, lung disease, BMI, hypertension and heart disease |  | treated DM and ≥1 complication -1.73 (0.61-4.89)  treated DM and ≥2 complications – 3.45 (1.59-7.50)  DCSI score <4 – 1.72 (0.72-4.13)  DCSI score ≥4 – 5.50 (2.11-12.04) |
| **Dobler et al, 2012** | Australia | Linkage of National Diabetes Service Scheme and state and territory TB notification databases General population comparison using census data from the Australian Bureau of Statistics.  271 TB cases in 802,087 DM cohort | 2001-2006 | Self-reported DM confirmed by health professional | Notification of active TB case, | Age, sex, indigenous status, TB incidence in country of birth.  Aggregate data available for general population cohort | RR 1.48 (1.04-2.10) | DM using insulin: RR 2.27 (1.41-3.66).  No evidence for interaction with age, indigenous status, sex or TB incidence in country of birth |
|  |  |  |  |  |  |  |  |  |
| **Table A5 continued** | | | | | | | | |
| **Study** | **Country** | **Population** | **Study period** | **Exposure** | **Outcome** | **Confounders adjusted for** | **RR/OR**  **(95% CI)** | **Additional analyses** |
| **Young et al, 2012** | England | Two consecutive Oxford Record Linkage Study (ORLS) datasets for hospital admissions and day-case care  ORLS1:  51 TB cases,  19,244 DM cohort  ORLS2:  12 TB cases  7943 DM cohort | ORLS1: 1963-1998  ORLS2: 1999-2005 | Diabetes recorded as main reason for admission to hospital | TB recorded as one of the admissions diagnoses | Stratified for age, sex, district and calendar year | ORLS1: mean follow-up 7.1 years; 1.83 (1.26-2.60)  ORLS2: mean follow-up 3.2 years; 3.11 (1.17-7.03) | Risk of DM after TB:  ORLS1: 1.12 (0.76-1.60) |
| **Kuo et al, 2013** | Taiwan | National Health Insurance medical claims database  5103 TB cases  253,369 total cohort | 2000-2011 | ICD-9-CM DM codes and continuous prescriptions for antidiabetic drugs for ≥60 days | ICD-9 codes and continuous prescriptions for anti-TB drugs for ≥60 days | Age, sex, asthma, bronchiectasis and COPD Controls matched on gender, year of birth and follow-up time | Mean DM follow-up 5 years  Mean non-DM follow-up 4.8 years  HR 1.31 (1.23-1.39) | Interaction between DM and asthma, bronchiectasis and COPD. HR 1.13 (1.01-1.26), 1.83 (1.47-2.26) and 1.25 (1.11-1.42) respectively |
|  |  |  |  |  |  |  |  |  |
|  |  |  |  |  |  |  |  |  |
|  |  |  |  |  |  |  |  |  |
|  |  |  |  |  |  |  |  |  |
|  |  |  |  |  |  |  |  |  |
|  |  |  |  |  |  |  |  |  |
|  |  |  |  |  |  |  |  |  |
| **Table A5 continued** | | | | | | | | |
| **Study** | **Country** | **Population** | **Study period** | **Exposure** | **Outcome** | **Confounders adjusted for** | **RR/OR**  **(95% CI)** | **Additional analyses** |
| **Case-control** | | | | | | | | |
| **Faurholt-Jepsen et al, 2011** | Tanzania | TB patients diagnosed at 4 health centres in Mwanza.  803 TB cases  350 consecutive neighbourhood controls | 2006-2009 | Fasting blood glucose or oral glucose tolerance test  DM testing few days after start of TB treatment. | Culture confirmed pulmonary TB. | Age, sex, socio-demography, HIV^7^ and acute phase protein alpha-1-acid glycoprotein. | Overall OR 2.2 (1.5-3.4)  Interaction with HIV status:  HIV +: OR 4.2  (1.5-11.6)  HIV -: OR 0.1 (0.01-1.8) | Prevalence HIV + 43.2% in TB cases |
| **Leegaard et al, 2011** | Denmark | First time TB diagnosis in Northern Denmark hospital records for residents aged ≥15 years  Danish Civil Registration System (DNRP) up to 5 matched controls  2,950 TB cases  14,274 matched controls  2,950 TB cases  14,274 controls | 1980-2008 | Three databases: DNRP, Aarhus University Prescription Database and Danish National Health Insurance Service Registry | First time TB diagnosis in hospital records | Conditions included in Charlson comorbidity index, alcoholism-related disorders, marital status, urbanization, marital status and number of children | Median follow-up 3.7 years  Overall OR 1.18 (0.96-1.45)  Type 1 DM: OR 2.59 (0.44-15.29)  No interaction with age, hba1c, calendar period or country of origin | Subset living in Jutland: corticosteroid, immunosuppressive, proton pump inhibitor data and Hba1c measurements  1,019 TB cases  4.856 controls.  Overall OR 1.02 (0.73-1.44)  Hba1c<7%: OR 0.91 (0.51-1.63),  Hba1c 7-7.9%: OR 1.05 (0.41-2.66), Hba1c ≥8%: OR 1.19 (0.61-2.30) |
| ^1^BMI: body mass index, ^2^ CVD: cardiovascular disease, ^3^HTN: hypertension, ^4^COPD: chronic obstructive pulmonary disease, ^5^ADL: activities of daily living and ^6^ICD-9-CM: International Classification of Disease, Ninth Revision, Clinical Modification. | | | | | | | | |
